# The Cholera Epidemic of 1873 in the United States

**Published:** 1875-10

**Authors:** 


*Cholera Epidemic of* 1873 *in the United States.*—Government Printing Office, Washington City.

This report is issued in accordance with the requirements of the joint resolution passed by both Houses of Congress, March 25th, 1874, authorizing an inquiry into and report upon the causes of epidemic cholera, derived for the Treasury and War Departments, and consists of one thousand and twenty-five pages.

